# Comparison of [^68^Ga]Ga-DOTA-FAPI-04 and [^18^F]FDG Uptake in Esophageal Cancer

**DOI:** 10.3389/fonc.2022.875081

**Published:** 2022-06-16

**Authors:** Huipan Liu, Zhi Hu, Xiao Yang, Tianyang Dai, Yue Chen

**Affiliations:** ^1^ Department of Nuclear Medicine, The Affiliated Hospital of Southwest Medical University, Luzhou, China; ^2^ Nuclear Medicine and Molecular Imaging Key Laboratory of Sichuan Province, Luzhou, China; ^3^ Institute of Nuclear Medicine, Southwest Medical University, Luzhou, China; ^4^ Academician (Expert) Workstation of Sichuan Province, Luzhou, China; ^5^ Department of Thoracic Surgery, The Affiliated Hospital of Southwest Medical University, Luzhou, China

**Keywords:** esophageal cancer, 68Ga-FAPI, PET/CT, lymph node metastasis, bone and visceral metastases

## Abstract

**Purpose:**

Accurate clinical staging is crucial to managing esophageal cancer. [^68^Ga]Ga-DOTA-FAPI-04 exhibits good diagnostic performance in various tumors, showing a promising alternative to [^18^F]FDG. Here, we investigated the diagnostic performance of [^68^Ga]Ga-DOTA-FAPI-04 PET/CT and [^18^F]FDG PET/CT in the diagnosis of primary and metastatic lesions of esophageal cancer.

**Methods:**

Patients with esophageal cancer who underwent concurrent [^68^Ga]Ga-DOTA-FAPI-04 and [^18^F]FDG PET/CT between January 2020 and June 2021 were retrospectively analyzed. [^68^Ga]Ga-DOTA-FAPI-04 and [^18^F]FDG PET/CT uptakes were compared by using the paired samples t test. The McNemar test was used to compare the diagnostic performance between the two techniques.

**Results:**

Thirty-five patients (ranging from 44-83 years old with a median age of 63.5 years) were evaluated in our study. In treatment-naive patients (n=25), [^68^Ga]Ga-DOTA-FAPI-04 PET could detect all esophageal cancers, whereas 1 patient with superficial esophageal cancer was negative in FDG but positive in [^68^Ga]Ga-DOTA-FAPI-04 (T1). [^68^Ga]Ga-DOTA-FAPI-04 uptake was higher than [^18^F]FDG in primary lesions (13.8 ± 6.9 vs 10.9 ± 6.8, respectively, P=0.004), involved lymph nodes (9.3 ± 5.2 vs 6.4 ± 5.9, respectively, P=0.002), and bone and visceral metastases (10.4 ± 6.0 vs 6.1 ± 7.5, respectively, P=0.001). In addition, [^68^Ga]Ga-DOTA-FAPI-04 PET/CT has a higher detection sensitivity than [^18^F]FDG PET/CT for primary tumors [100% (25/25) vs. 96.0% (24/25), respectively], lymph nodes [95.0% (57/60) vs 75.0% (45/60), P<0.001], and bone and visceral metastases [100% (25/25) vs 72% (18/25), respectively; P= 0.008].

**Conclusion:**

[^68^Ga]Ga-DOTA-FAPI-04 PET/CT has higher tracer uptake value and is superior to [^18^F]FDG PET/CT in detecting primary and metastatic lesions in patients with esophageal cancer.

## Introduction

With approximately 600,000 new diagnoses and 540,000 deaths in 2020, esophageal cancer is the sixth leading cause of cancer-related death worldwide (1). Squamous cell carcinoma is the predominant histological type of esophageal cancer in East Asia, East and Southern Africa, and Southern Europe, while adenocarcinoma is most common in Northern and Western Europe, Oceania, and North America. Fibroblast activating protein (FAP) is overexpressed in cancer-associated fibroblasts (CAF) of various epithelial cancers, and at levels in normal tissues (2, 3). Therefore, imaging targeting FAP is considered a promising strategy for visualization of the tumor stroma, which is mainly composed of CAFs (4). Gallium-68 (^68^Ga)-labeled FAP inhibitor ([^68^Ga]Ga-DOTA-FAPI-04) is a promising PET tracer for imaging a variety of tumors (2, 4, 5). Studies have shown that [^68^Ga]Ga-DOTA-FAPI-04 enables fast imaging, showing exceptionally sharp tumor outlines and high tumor-to-background contrast in a variety of tumors (2, 4). In addition, case studies reported the application of [^68^Ga]Ga-DOTA-FAPI-04 PET/CT in the detection of primary tumors and metastatic lymph nodes in esophageal cancer (5–7). However, the exact diagnostic utility of [^68^Ga]Ga-DOTA-FAPI-04 in esophageal cancer has not been systematically analyzed.

Here, we retrospectively compared the potential efficacy of [^68^Ga]Ga-DOTA-FAPI-04 PET/CT with [^18^F]FDG PET/CT in the diagnosis of primary and metastatic lesions in patients with esophageal cancer.

## Materials and Methods

### Patients

This retrospective study was conducted at the Affiliated Hospital of Southwest Medical University from January 2020 to June 2021. This study was approved by the Ethics Committee of our hospital (AHSWMU-2020-035) and informed consent was obtained from each patient. The inclusion criteria were as follows: (I) patients with newly diagnosed esophageal cancer or esophageal cancer after surgical resection, (II) patients aged 18 years or older, (III) patients who agreed to undergo both [^18^F]FDG and [^68^Ga]Ga-DOTA-FAPI-04 PET/CT scans for comparison, (IV) patients participating in this study were able to provide written informed consent. Exclusion criteria were as follows: (I) pregnant patients, (II) patients with newly diagnosed esophageal cancer who have started treatment before [^68^Ga]Ga-DOTA-FAPI-04 PET/CT examination, (III) patients unable or unwilling to provide written informed consent. In our study, histopathological examination of biopsy or excisional surgical specimens or follow-up served as the reference standard for final diagnosis.

### Preparation of [^18^F]FDG and [^68^Ga]Ga-DOTA-FAPI-04

[^18^F]FDG was manufactured in accordance with the standard method using the coincidence [^18^F]FDG synthesis module (FDG-N, PET Science & Technology, Beijing, China). We purchased the precursor FAPI-04 from MCE (MedChemExpress, USA), with a purity of 98% and a quality of 872.91. The [^68^Ga]Ga-DOTA-FAPI-04 labeling was carried out according to the method described previously (8, 9). The radiochemical purity of [^68^Ga]Ga-DOTA-FAPI-04 and [^18^F]FDG exceeded 95%. The sterility test was carried out by the radiochemical equipment of our department. The final product was sterile and met all of our institution’s required standards prior to use.

### PET/CT Imaging

For [^18^F]FDG PET/CT image acquisition, patients fasted for at least 6 hours and the plasma glucose level was lower than 11 mmol/L (about 200 mg/dL) (10). After an intravenous injection of [^18^F]FDG 3.7 MBq/kg, the patient rested in a quiet place. The patients were instructed to drink 500 mL of water to stimulate [^18^F]FDG excretion in the renal calyx and to urinate before scanning. Data were acquired using a PET/CT scanner (uMI780, United Imaging, Shanghai, China) 45 to 60 minutes after intravenous administration. First, a CT scan was performed from the head to the upper thigh (tube current of 120 mA, tube voltage of 120 kV, and slice thickness of 3.00 mm). PET was then performed at the same bed position as the CT scan, 5-6 bed positions, and 3D acquisition mode at 1.5 min/position. [^68^Ga]Ga-DOTA-FAPI-04 PET/CT was performed within 7 days of [^18^F]FDG PET/CT for comparison and the dose of [^68^Ga]Ga-DOTA-FAPI-04 injected was calculated based on patient body weight (1.85 MBq/kg). Other parameters of CT or PET were the same as [^18^F]FDG PET/CT, except that [^68^Ga]Ga-DOTA-FAPI-04 PET imaging was acquired at 3.0 min/position. After the reconstruction was complete, image analysis was performed using the joint imaging post-processing fusion software.

### PET/CT Imaging Review

Both [^18^F]FDG and [^68^Ga]Ga-DOTA-FAPI-04 PET/CT images were interpreted in random order by two experienced nuclear medicine physicians. Any differences of opinion would be resolved by consensus. Based on knowledge of the normal biodistribution of [^18^F]FDG and [^68^Ga]Ga-DOTA-FAPI-04, lesions were identified as positive lesions with increased tracer uptake in comparable normal contralateral structures and surrounding soft tissue. Lesions were characterized as either positively or possibly abnormal (i.e., representing a tumor) if the uptake of [^18^F]FDG and [^68^Ga]Ga-DOTA-FAPI-04 was markedly to moderately increased. Diffuse mild or no increase in activity (in the absence of an abnormality on CT and no corresponding abnormality on PET) was considered normal or benign disease. PET/CT results were divided into primary tumor, lymph node metastasis, and distant metastasis. The individual lymph nodes were then divided into four regions including the neck, upper mediastinum, lower mediastinum, and abdomen. Distant involvement, such as lung, liver, bone, pleura, and brain metastases were each classified as a separate site. The largest lesion was measured according to length for individual primary tumor, each lymph node region, and distant involvement site. For [^18^F]FDG and [^68^Ga]Ga-DOTA-FAPI-04 PET/CT, the number of lesions per lymph node region or distant metastatic site and the SUVmax of the lesions with the highest tracer accumulation were recorded.

### Reference Standard

[^68^Ga]Ga-DOTA-FAPI-04 and [^18^F]FDG PET/CT findings were validated by cytology/histopathology as the gold standard. In the case of the absence of histopathological correlation, clinical and radiologic follow-up findings, up to at least 3 months, were taken into consideration to validate the [^68^Ga]Ga-DOTA-FAPI-04 and [^18^F]FDG PET/CT findings.

### Statistical Analysis

All statistical analyses were performed using SPSS software (version 22.0; IBM, Armonk, NY). Paired samples t-test was used to compare the SUVmax of primary tumors, lymph node metastasis and distant metastases of [^18^F]FDG and [^68^Ga]Ga-DOTA-FAPI-04 PET/CT. Results from the visually interpreted PET/CT images were compared with histopathology or follow-up results. We compared the statistical differences in detection rates of primary tumors, lymph nodes, and visceral metastases by [^18^F]FDG and [^68^Ga]Ga-DOTA-FAPI-04 PET/CT using the McNemar test. Sensitivity, specificity, accuracy, positive predictive value, and negative predictive value for the diagnosis of [^18^F]FDG and [^68^Ga]Ga-DOTA-FAPI-04 were calculated and compared using McNemar’s test to evaluate the diagnostic effect. P<0.05 was considered to indicate a statistically significant difference.

## Results

### Patient Characteristics

Between January 2020 and June 2021, 35 patients (32 men, 3 women, median age: 63.5 years old, ranging from 44–83 years) were included in this study. Among these patients, 34 had squamous carcinoma and 1 had adenocarcinoma. Also, 25 patients underwent PET/CT for initial tumor evaluation and 10 patients underwent PET/CT for post-operative recurrence detection. Patient characteristics are summarized in **Table 1**.

**Table 1:** Summary of Patient Characteristics.

Characteristic	Value
Number of patients	35
Age (year-old)	
Median	63.5
Range	44-83
Sex	
Men	32
Women	3
Indication for PET	
Initial assessment (staging)	25
Recurrence detection (restaging)	10
Patient status	
Treatment-naive	25
Resection surgery	3
Chemotherapy after surgery	3
Chemoradiotherapy after surgery	3
Targeted therapy and chemotherapy after surgery	1
Histologic findings	
Squamous carcinoma	34
Adenocarcinoma	1

### Comparative Results for Initial Assessment and Recurrence Detection

#### Detection of Primary Cancer

**Figure 1:**
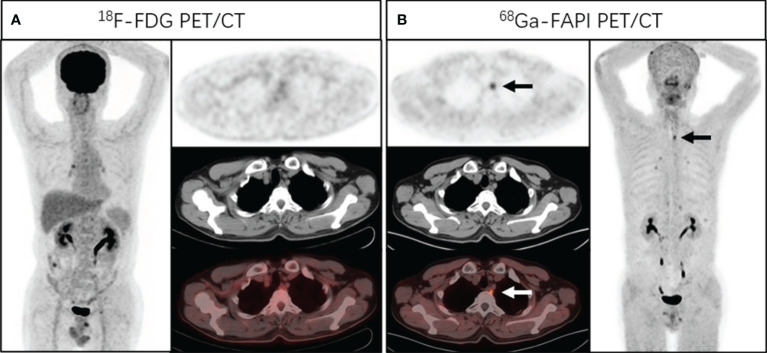
A 58-year-old man presented with a complaint of progressive dysphagia and back pain for 2 months. Endoscopic biopsy demonstrated a well-differentiated squamous cell carcinoma of the upper esophagus. Chest CT and [^18^F]FDG PET/CT **(A)** showed no abnormal density and activity in esophagus. [^68^Ga]Ga-DOTA-FAPI-04 PET/CT **(B)** revealed a focus of intensive uptake in the upper esophageal cancer (arrows, SUVmax of 3.4); no other foci of [^68^Ga]Ga-DOTA-FAPI-04 uptake associated with esophageal cancer were seen. Subsequently, the patient received radical radiotherapy plus chemotherapy.

**Figure 2:**
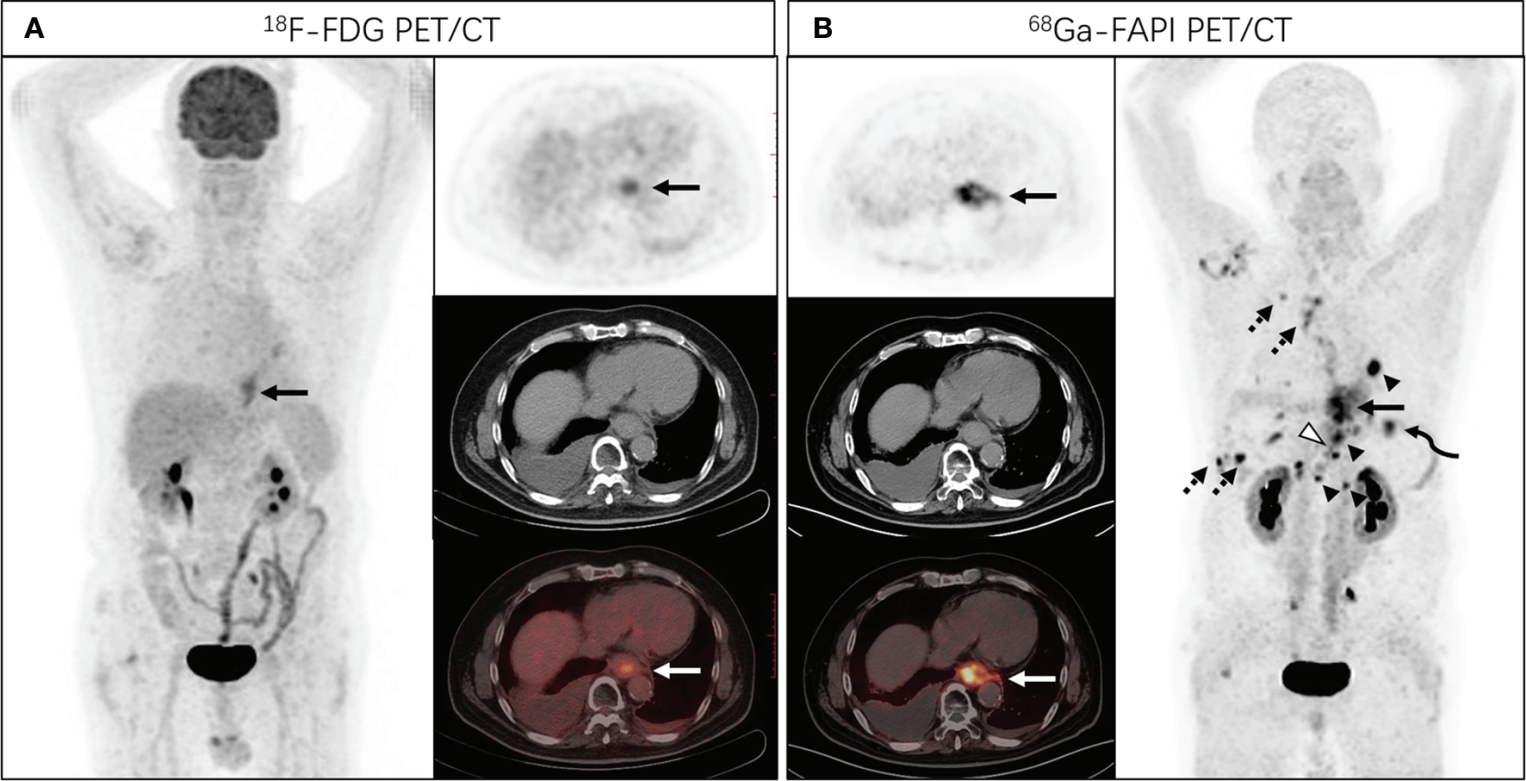
A 68-year-old man presented with a complaint of progressive dysphagia for 3 months. Endoscopic biopsy demonstrated a poorly differentiated adenocarcinoma of the lower esophagus. [^18^F]FDG PET/CT **(A)** showed increased tracer uptake in lower esophagus (SUVmax of 5.7), and no distant metastasis was identified. Subsequent [^68^Ga]Ga-DOTA-FAPI-04 PET/CT **(B)** revealed intense FAPI uptake in the distal esophagus with SUVmax of 15.2 (solid arrows), with nodal metastases (arrowheads, SUVmax of 21.8), bone (hollowed arrowheads, SUVmax of 14.2), lung (curved arrowheads, SUVmax of 11.0) and pleural metastases (dotted arrows, SUVmax of 15.5).

Of the 25 patients initially evaluated, 1 patient was negative in [^18^F]FDG PET/CT, but positive in [^68^Ga]Ga-DOTA-FAPI-04 PET/CT, which was T1 (superficial esophageal cancer) staging in biopsy (**Figure 1**). For individual primary tumor analysis (n=25), the SUVmax of [^68^Ga]Ga-DOTA-FAPI-04 PET was significantly higher than [^18^F]FDG (13.8 ± 6.9 vs 10.9 ± 6.8, P=0.004). The true-positive rates for [^18^F]FDG PET/CT and [^68^Ga]Ga-DOTA-FAPI-04 PET/CT were 96.0% (24 of 25) and 100% (25 of 25), respectively. Regarding the 10 patients with recurrence detection after surgery, the true-positive rates for [^18^F]FDG PET/CT and [^68^Ga]Ga-DOTA-FAPI-04 PET/CT were 100% (6 of 6) and 100% (6 of 6), respectively. The detailed comparative results of detection are shown in **Table 2**. **Figure 2** shows comparison of [^18^F]FDG and [^68^Ga]Ga-DOTA-FAPI-04 PET/CT.

**Table 2:** Comparison of [^18^F]FDG and [^68^Ga]Ga-DOTA-FAPI-04 Uptake in Esophageal cancer.

Parameter	Tumor Size (cm)	[^18^F]FDG Uptake	Number of PositiveLesions	[^68^Ga]Ga-DOTA-FAPI-04 Uptake	No. of Positive Lesions	P Value
**Primary tumor**	4.3 ± 2.2	10.9 ± 6.8	24	13.8 ± 6.9	25	0.004
**Involved lymph nodes**						
Neck and supraclavicular	1.6 ± 1.2	6.0 ± 5.5	11	10.9 ± 7.1	13	0.040
Upper Mediastinum	1.4 ± 1.0	5.5 ± 4.5	13	8.2 ± 4.0	18	0.033
Lower Mediastinum	0.9 ± 0.3	5.7 ± 6.0	9	7.6 ± 4.8	13	0.356
Abdomen	2.0 ± 1.3	9.3 ± 8.0	10	10.9 ± 4.3	11	0.337
All	1.5 ± 1.1	6.4 ± 5.9	45	9.3 ± 5.2	57	0.002
**Bone and visceral metastasis**						
Bone	1.0 ± 0.6	9.3 ± 9.3	8	12.9 ± 7.4	11	0.072
Pleural	0.8 ± 0.3	2.7 ± 3.5	3	9.2 ± 4.0	7	0.046
Lung	0.6 ± 0.1	3.0 ± 2.9	3	6.6 ± 4.4	3	0.425
Liver	1.6	15.4	1	12.6	1	NA
Subcutaneous metastasis	1.3	5.8	1	12	1	NA
Adrenal glands	1.2 ± 0.3	7.3 ± 0.2	2	4.7 ± 1.6	2	0.281
All	1.3 ± 0.4	6.1 ± 7.5	17	10.4 ± 6.0	25	0.001

NA, not applicable

#### Detection of Nodal Metastasis

Among the 35 patients, 60 lymph node metastases were examined in 20 patients and 57 of the 60 lymph nodes were correctly identified with [^68^Ga]Ga-DOTA-FAPI-04 for lymph node involvement (false-positive uptake in 2 lymph nodes and false-negative uptake in 3 lymph nodes). In contrast, 45 of the 60 lymph node metastases were correctly diagnosed by [^18^F]FDG PET/CT (false-positive uptake in 29 lymph nodes and false-negative uptake in 15 lymph nodes).

In the nodal region-based analysis, 12 lymph node metastases were not detected by [^18^F]FDG but revealed by [^68^Ga]Ga-DOTA-FAPI-04 PET/CT; 27 lymph nodes were misdiagnosed as lymph node metastases by [^18^F]FDG PET but showed no [^68^Ga]Ga-DOTA-FAPI-04 uptake; and 2 lymph nodes were misdiagnosed as lymph node metastases by positive [^68^Ga]Ga-DOTA-FAPI-04 uptake, which were finally confirmed as lymphnoditis. In addition, both [^18^F]FDG and [^68^Ga]Ga-DOTA-FAPI-04 showed 2 false-negative lymph nodes which were paraesophageal lymph nodes.

For all 60 nodal metastases, [^68^Ga]Ga-DOTA-FAPI-04 PET showed significantly higher SUVmax value than [^18^F]FDG PET (9.3 ± 5.2 vs 6.4 ± 5.9, P=0.002). The sensitivity, specificity, accuracy, PPV, and NPV of detecting metastatic lymph nodes were 75.0%, 77.2%, 76.4%, 60.8% and 86.7% for [^18^F]FDG PET/CT and 95.0%, 98.4%, 97.3%, 96.6% and 97.6% for [^68^Ga]Ga-DOTA-FAPI-04 PET/CT, respectively (**Table 3**).

**Table 3:** Diagnostic Performance of [^68^Ga]Ga-DOTA-FAPI-04 and [^18^F]FDG PET/CT in Assessment of Lymph Node Metastases.

Study type	Neck	Upper Mediastinal	Lower Mediastinal	Abdomen	Total
[^18^F]FDG					
sensitivity	80.0	65.0	71.4	90.9	75.0
specifcity	66.7	90.0	64.2	100	77.2
accuracy	77.8	81.7	65.4	96.4	76.4
PPV	92.3	76.5	29.4	100	60.8
NPV	40.0	83.7	91.4	94.4	86.7
[^68^Ga]Ga-DOTA-FAPI-04					
sensitivity	93.3	90.0	100	100	95.0
specifcity	100	100	97.0	100	98.4
accuracy	94.4	96.7	97.5	100	97.3
PPV	100	100	87.5	100	96.6
NPV	75.0	95.2	100	100	97.6

In comparison, [^68^Ga]Ga-DOTA-FAPI-04 PET/CT showed a higher detection efficacy for metastatic lymph node compared to [^18^F]FDG PET/CT (P<0.001).

#### Detection of Distant Metastasis

A total of 6 different distant sites of involvement and 25 metastases were identified in 35 patients according to the gold and reference standards. [^68^Ga]Ga-DOTA-FAPI-04 PET/CT detected all of these lesions and 25 of 25 metastases were correctly identified. However, [^18^F]FDG PET/CT missed 7 metastatic lesions (false-negative uptake in 3 bone metastases and 4 pleural metastases). [^68^Ga]Ga-DOTA-FAPI-04 PET had significantly higher SUVmax values than [^18^F]FDG PET based on all metastatic lesions analyzed (10.4 ± 6.0 vs 6.1 ± 7.5, P=0.001). The sensitivity of [^68^Ga]Ga-DOTA-FAPI-04 PET/CT was better than that of [^18^F]FDG PET/CT [100% (25 of 25) vs 72% (18 of 25), respectively; P= 0.008]. Biopsy confirmation of all suspicious lesions for the sole purpose of validating PET/CT results was not ethical. Thus, neither a true-negative nor a false-positive state in these participants could be accurately established.

## Discussion

[^18^F]FDG PET is a metabolic imaging technique that provides information preceded by structural changes (11). [^18^F]FDG is more sensitive than CT in detecting metastatic disease and is now widely used for preoperative staging of tumor patients. FAP is a type II transmembrane serine protease, and [^68^Ga]Ga-DOTA-FAPI-04 is used for imaging tumor stromal (2). Compared to [^18^F]FDG, [^68^Ga]Ga-DOTA-FAPI-04 shows several advantages, including equal or better tumor-to-background ratio, independence of blood glucose levels, rapid renal clearance, and feasibility of rapid image acquisition (12, 13). [^18^F]FDG PET has, instead, mainly been used for advanced cancer and is not indicative in staging superficial esophageal cancer due to spatial resolution limitations (14). This study has shown that [^68^Ga]Ga-DOTA-FAPI-04 was able to visualize small tumors (diameter < 1.0 cm) which might be missed by the [^18^F]FDG PET/CT (4). It was conducted on 25 patients of initial assessment and showed that [^68^Ga]Ga-DOTA-FAPI-04 PET could detect all esophageal cancers, whereas 1 patient with superficial esophageal cancer was negative in [^18^F]FDG but positive in [^68^Ga]Ga-DOTA-FAPI-04 (T1). [^68^Ga]Ga-DOTA-FAPI-04 seems to be more advantageous for early esophageal cancer than [^18^F]FDG. Furthermore, [^68^Ga]Ga-DOTA-FAPI-04 and [^18^F]FDG exhibited the same detection performance in recurrence detection. Therefore, [^68^Ga]Ga-DOTA-FAPI-04 may also have a good application prospect for postoperative patient monitoring.

The overall 5-year survival rate for patients with esophageal cancer is approximately 40% (15). The presence or absence of lymph node metastases is one of the most important prognostic factors, however, because the location of metastatic lymph nodes is often variable, identification is difficult (16). Regarding the detection of lymph nodes, lymph node staging in patients with esophageal cancer is critical for treatment and prognosis. To our knowledge (17, 18), due to the activation of inflammatory cells, non-specific lymph node inflammation will cause increased [^18^F]FDG uptake. Additionally, the main limitation of [^18^F]FDG PET/CT in staging of esophageal cancer is its low to moderate sensitivity for lymph node staging and delineation between viable tumor and regional esophagitis (19). The false-positive rate of [^18^F]FDG was the highest in the subcarinal and bronchus lymph nodes (15). In our study, [^68^Ga]Ga-DOTA-FAPI-04 PET/CT showed more positive lymph nodes in the neck, mediastinum, and abdomen than [^18^F]FDG PET/CT, and the false positive rate of [^68^Ga]Ga-DOTA-FAPI-04 is relatively lower than [^18^F]FDG in lymph nodes, especially in mediastinal lymph nodes. In lymph node metastases, the uptake of [^68^Ga]Ga-DOTA-FAPI-04 was higher than that of [^18^F]FDG. Moreover, due to the high uptake of radiotracer in the primary lesion, [^18^F]FDG PET/CT has limited value for detection of paraesophageal lymph nodes. This seems to still exist in [^68^Ga]Ga-DOTA-FAPI-04. Nevertheless, [^68^Ga]Ga-DOTA-FAPI-04 PET/CT may be more sensitive than [^18^F]FDG in detecting metastatic lymph nodes in patients with esophageal cancer, which helps to accurately guide clinicians to determine reasonable treatment options.

In detecting bone and visceral metastases, [^68^Ga]Ga-DOTA-FAPI-04 PET/CT detected more abnormal, bone, and pleural lesions than [^18^F]FDG PET/CT. The sensitivity of [^68^Ga]Ga-DOTA-FAPI-04 PET/CT in detecting bone metastases and visceral metastases was significantly higher than that of [^18^F]FDG PET/CT. Our study shows that [^68^Ga]Ga-DOTA-FAPI-04 is superior to [^18^F]FDG PET/CT in detecting pleural metastases, even small pleural metastatic lesions (<1.0 cm in diameter). [^68^Ga]Ga-DOTA-FAPI-04 PET/CT detected all distant lesions, and 25 out of 25 distant metastases were correctly identified. However, [^18^F]FDG PET/CT missed 7 metastases (3 bone metastases and 4 pleural metastases with false negative uptake). The accurate diagnosis of bone metastases and pleural metastases by [^68^Ga]Ga-DOTA-FAPI-04 PET/CT can help guide subsequent clinical oncological management.

Our study also has limitations. First, the patient sample size was relatively small (n=35) and a prospective trial with a larger patient population is required to further investigate the diagnostic performance of [^68^Ga]Ga-DOTA-FAPI-04 PET/CT. Furthermore, not all lesions were histopathologically confirmed.

## Conclusion

Our findings demonstrate that [^68^Ga]Ga-DOTA-FAPI-04 PET/CT has higher tracer uptake value and is superior to [^18^F]FDG PET/CT in detecting primary and metastatic lesions in patients with esophageal cancer.

## Data Availability Statement

The original contributions presented in the study are included in the article/supplementary material. Further inquiries can be directed to the corresponding author.

## Ethics Statement

This retrospective study was obtained approval from the Ethics Committee of the Affiliated Hospital of Southwest Medical University (AHSWMU-2020-035). The patients/participants provided their written informed consent to participate in this study.

## Author Contributions

All authors conceived and designed the study. HL conducted statistical analyses; all authors interpreted the findings. HL, ZH, and XY drafted the manuscript. TD and YC provided critical review of the manuscript for key intellectual content. YC was the guarantors, and as such, had full access to the data and take responsibility for its integrity and accuracy. All authors approved the final manuscript.

## Conflict of Interest

The authors declare that the research was conducted in the absence of any commercial or financial relationships that could be construed as a potential conflict of interest.

## Publisher’s Note

All claims expressed in this article are solely those of the authors and do not necessarily represent those of their affiliated organizations, or those of the publisher, the editors and the reviewers. Any product that may be evaluated in this article, or claim that may be made by its manufacturer, is not guaranteed or endorsed by the publisher.
